# Hypomagnesemia Is Associated with Excessive Daytime Sleepiness, but Not Insomnia, in Older Adults

**DOI:** 10.3390/nu15112467

**Published:** 2023-05-25

**Authors:** Muhammed Tunc, Pinar Soysal, Ozge Pasin, Lee Smith, Masoud Rahmati, Veliye Yigitalp, Sevnaz Sahin, Moustapha Dramé

**Affiliations:** 1 Division of Internal Medicine, Faculty of Medicine, Bezmialem Vakif University, Istanbul 34093, Türkiye; mtunc@bezmialem.edu.tr; 2 Division of Geriatric Medicine, Faculty of Medicine, Bezmialem Vakif University, Istanbul 34093, Türkiye; yigitalpveliye@gmail.com; 3 Division of Biostatistics, Faculty of Medicine, Bezmialem Vakif University, Istanbul 34093, Türkiye; ozgepasin90@yahoo.com.tr; 4 Centre for Health, Performance, and Wellbeing, Anglia Ruskin University, Cambridge CB1 1PT, UK; lee.smith@aru.ac.uk; 5 Division of Physical Education and Sport Sciences, Faculty of Literature and Human Sciences, Lorestan University, Khoramabad 68151-44316, Iran; rahmati.mas@lu.ac.ir; 6 Division of Geriatrics, Department of Internal Medicine, Faculty of Medicine, Ege University, Izmir 35040, Türkiye; drsevnaz@gmail.com; 7 Division of Clinical Research and Innovation, University Hospitals of Martinique, 97261 Fort-de-France, France; moustapha.drame@chu-martinique.fr

**Keywords:** excessive daytime sleepiness, hypomagnesemia, insomnia, elderly

## Abstract

The aim of this study was to investigate associations between serum magnesium levels with insomnia and excessive daytime sleepiness (EDS) in older adults. A total of 938 older outpatients were included in the study. Hypomagnesemia was defined as serum magnesium concentration below <1.6 mg/dL. Patients were divided into two groups: hypomagnesemia and normomagnesia (1.6–2.6 mg/dL). The Epworth Sleepiness Scale was implemented and scores of ≥11 points were categorized as EDS. The Insomnia Severity Index was implemented and scores of ≥8 indicated insomnia. The mean age was 81.1 ± 7.6 years. While the presence of EDS, hypertension, diabetes mellitus, and coronary artery disease were more common in the hypomagnesemia group than the normomagnesia group, Parkinson’s disease was less common (*p* < 0.05). Hemoglobin and HDL cholesterol were lower, whereas HbA1c, triglyceride, and number of drugs used were higher in the hypomagnesemia group compared to the normomagnesia group (*p* < 0.05). In both univariate analysis and multivariate analysis adjusted for gender, age and all confounders, there were significant associations between hypomagnesemia and EDS [odds ratio (OR):1.7; 95% confidence interval (CI): 1.6–2.6, and OR: 1.9; 95%CI: 1.2–3.3, respectively (*p* < 0.05)]. There was no significant relationship between hypomagnesemia and insomnia (*p* > 0.05). The present study identified an association between hypomagnesemia and EDS in older adults. Therefore, it may be prudent to consider hypomagnesemia when evaluating older adults with EDS and vice versa.

## 1. Introduction 

Magnesium plays a role in the maintenance of vascular tone, thrombus formation, cardiac conduction, and neurotransmitter synthesis, as well as acting as a cofactor in many enzymatic reactions [1]. Hypomagnesemia, which is associated with chronic diseases, gastrointestinal system or renal loss, and low intake or alcohol use, is often neglected in clinical practice [1,2]. With aging, decreases in the total level of magnesium occur mainly owing to a decrease in oral magnesium intake as a result of consumption of less green vegetables, which are key sources of magnesium [3,4]. Decreased intestinal reabsorption of magnesium, increased urinary excretion, and drug interactions also contribute to magnesium deficiency in the geriatric population [2]. 

Magnesium is an essential element required for the regulation of various cellular and metabolic reactions, including ATP generation, DNA replication, and DNA repair. It is an important co-factor for the folate–methionine–neurotransmitter cycle; and thus, magnesium has a pivotal role in the neurotransmitter synthesis pathway [5]. The disruption of methylation processes leads to a build-up of homocysteine, thereby increasing the likelihood of inflammation, oxidative stress, and subsequent damage to mitochondria and DNA [6,7]. Additionally, telomeres are protective nucleoprotein structures at the ends of all chromosomes that provide genomic stability and prevent the loss of coding DNA, and impaired telomeric structure has a sensitivity to oxidative damage. The telomeric chromatin structure and integrity is impacted upon by magnesium biochemistry [8]. The effect of magnesium on cellular aging may be related to its interactions with telomere homeostasis, telomere maintenance, and activity of telomerase. A recent study showed that inadequate magnesium levels have an adverse impact on telomere attrition rate in older people with sleep disturbances [9]. Moreover, magnesium may also enhance melatonin secretion which promotes sleep onset [10]. Magnesium is important for the synthesis of N-acetyltransferase, which converts 5-hydroxytryptamine into N-acetyl-5-hydroxytryptamine, which can then be converted to melatonin [9]. Magnesium also contributes to the maintenance of a normal circadian rhythm and sleep quality and thus may reduce insomnia.

Findings from analyses of The National Health and Nutrition Examination’s database have found that aging is a superposed risk factor for deficient magnesium consumption and that magnesium consumption has a progressive decrease with age [11]. Indeed, the literature has shown that intestinal magnesium absorption and bio-variability vary with age [12]. The change in intestinal absorption of magnesium in older people is usually exacerbated by the disruption of vitamin D homeostasis. Renal magnesium reabsorption is an active process that occurs in the loop of Henle and the proximal convoluted tubule. Decreased kidney functionality, which is also common in old age, is likely to be an additional cause of magnesium loss [12,13]. In epidemiological and clinical studies, magnesium deprivation has been associated with low serum magnesium levels. Moreover, low dietary magnesium intake has been found to be associated with low-grade systemic inflammation, increased levels of inflammatory markers, proinflammatory molecules, and increased production of free oxygen radicals [13]. Ageing is accompanied by a low-grade inflammatory condition called “inflammaging”. A chronic magnesium deficiency and disruption of the redox state that expedites this inflammatory condition may have a significant impact on the development of age-related diseases and geriatric syndromes [2]. Moreover, magnesium acts as a crucial cofactor for both acquired and innate immune responses and is implicated in pathways that control the development and maintenance of immune cell activation and homeostasis [14]. There may be a relationship between magnesium deficiency and the condition of insulin resistance, T2DM, the emergence of the cardiometabolic syndrome, and prognosis of infectious clinical course [13]. Therefore, it can be hypothesized that maintaining an optimal magnesium balance throughout life may help prevent inflammation and related conditions associated with magnesium deficiency, thereby helping to prolong healthy life.

There are limited studies on the prevalence of hypomagnesemia in the elderly. However, a small number of studies have reported that the prevalence of hypomagnesemia is between 20–25% in the general population, and over 50% in hospitalized patients [15,16,17]. Although hypomagnesemia is common, clinical symptoms or signs are often asymptomatic or are non-specific presentations, which can include fatigue, weakness, dizziness, depressive mood, anxiety, hyperemotionality, sleep disturbances, headache, myalgias, non-specific pain, and cramps. Severe hypomagnesemia can be more symptomatic, with presentations including muscle fasciculation, orthostatic hypotension, tremor, dysphagia, presence of Trousseau’s sign and Chvostek’s sign, and hypertension [18]. Chronic hypomagnesemia is often clinically undiagnosed, but it has many long-term negative outcomes [13]. It has been observed that hypomagnesemia is associated with an increased risk of delirium, geriatric depression, psychiatric disorders, Alzheimer’s disease, other dementia, and mortality in the elderly [13,19].

One important implication of magnesium deficiency is subsequent sleep complications. Sleep disorders are a common health problem that negatively affect quality of life and functionality in older people [15]. Indeed, magnesium is considered to have important effects on sleep regulation and circadian rhythm as it is a natural antagonist of N-metil-D-aspartic acid (NMDA) receptors and an agonist of gamma-aminobutyric acid (GABA) receptors, as well as having important effects on the regulation of the central nervous system [20]. It is likely that magnesium acts as a relaxant and anti-depressant. Therefore, magnesium may increase melatonin and renin levels as well as reduce levels of cortisol [20]. Recent research has found that magnesium may play a crucial role in the regulation of cellular timekeeping, energy balance, and circadian rhythm, and consequently sleep regulation [20]. However, despite this, study findings on the relationship between magnesium levels and sleep quality or quantity are inconsistent. For example, in one study carried out on a sample of adults, dietary magnesium intake was found to be significantly higher in those with adequate sleep quality in comparison to those without [21]. In another study, it was observed that magnesium had no superiority on sleep quality over placebo [22]. Although magnesium supplements have been shown to have a positive effect on insomnia parameters such as sleep efficiency, sleep time and sleep onset latency, and early morning awakening in the elderly [23], to date, there is no study in which hypomagnesemia and insomnia and excessive daytime sleepiness (EDS), two common sleep disorders in the elderly, have been evaluated simultaneously.

Therefore, the aim of this study was to investigate whether there is a relationship between hypomagnesemia and insomnia or EDS in the elderly.

## 2. Materials and Methods

In the retrospective monocentric study, patients aged 60 years and older who applied to one University Hospital Geriatrics outpatient clinic in Türkiye between December 2018 and January 2023 were retrospectively screened. The patients were evaluated according to inclusion and exclusion criteria. The local Ethics in Research Committees of the institutes approved the study.

### 2.1. Inclusion Criteria

**Figure 1:**
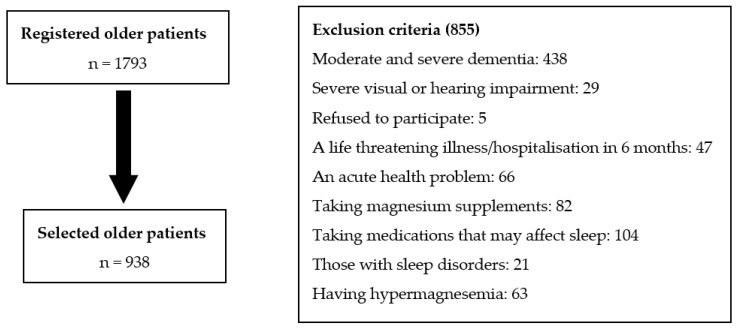
Study flow chart.

Accepting detailed geriatric evaluation: The patients who underwent insomnia severity index and Epworth Sleepiness Scale, and the patients whose file records were not incomplete and whose serum magnesium levels were checked on the same day, were included in our study. All patients who did not have an exclusion criterion were included in this study (Figure 1).

### 2.2. Exclusion Criteria

Those with moderate and severe dementia, those with severe visual or hearing impairment that prevented communication and understanding commands during the examination, those who refusde to participate in the examination, those who had a fatal illness, those who had a life-threatening illness in the last 6 months, or those who had been hospitalized for a major surgery were excluded from the present study. In addition, those who were determined to have an acute health problem (such as acute kidney failure, delirium, or stroke); those taking magnesium supplements or taking medications that may affect sleep, such as trazadone, mirtazapine, melatonin, antipsychotics, benzodiazepines, methylphenidate, and modafinil; and those with sleep disorders, such as sleep apnea syndrome, or central disorders of hypersomnolence, such as narcolepsy or restless leg syndrome, were also excluded. Those who were detected to have hypermagnesemia (≥2.6 mg/dL) according to serum magnesium values were also excluded [24].

### 2.3. Compherensive Geriatric Assessment [14]

Patients were assessed by a geriatrician whereas study data were collected by a gerontologist. The following variables were reported: participant age in years, sex, education status, marital and living status, number of medications, and chronic comorbid diseases (hypertension, diabetes mellitus, chronic obstructive pulmonary disease, coronary artery disease, congestive heart disease, dementia, periferic artery disease, cerebrovascular event, Parkinson’s disease, and osteoarthritis).

The Barthel Index (BADL) was used to evaluate the patients’ basic activities of daily living, and the Lawton index was used to evaluate instrumental activities of daily living (IADL). BADL includes functional status and the level of independence in basic daily living activities for feeding, bathing, dressing, bowel or bladder control, using the toilet, transfers, mobility, and stair climbing. A total score of 100 indicates full independence, whereas a score of 0 shows dependency on another person. The IADL measures abilities, including food preparation, shopping, handling finances, the use of a telephone, taking own medication, laundry, transportation and housekeeping. A total score of 23 indicates complete independence, whereas a score of 0 shows complete dependency on another person for IADL [25]. 

Information on nocturia was collected and nocturia was defined by the International Continence Society [26]. “Overall, in the last 30 days, how many times have you usually urinated from going to bed to waking up in the morning?” Urination at least once a night was considered as nocturia. The mean number of nocturia episodes of the patients was recorded [26]. Urinary incontinence was considered to be present in the individuals who had involuntary urinary incontinence in the last 3 months, except in cases without urinary tract infection and similar temporary conditions. Depression was diagnosed using the Geriatric Depression Scale-15. A score of ≥5 on the Geriatric Depression Scale-15 was classified as depression [27]. 

### 2.4. Evaluation of Insomnia

The Insomnia Severity Index includes seven self-reported items. These items assess symptoms of insomnia as well as the daytime impact, and were designed in accordance with criteria from the Diagnostic and Statistical Manual of Mental Disorders, Fifth Edition. The Insomnia Severity Index scores range from 0 (no insomnia) to 28 (severe insomnia). The Insomnia Severity Index scores for the present study were categorised as follows: mild (14–19), moderate (20–26), and severe (22–28) [28].

### 2.5. Evaluation of Excessive Daytime Sleepiness

The Epworth Sleepiness Scale was employed to examine EDS. The Epworth Sleepiness Scale is composed of eight items and the participant self-reports responses on a 4-point Likert scale. The patient is asked to report the possibility of “napping” while watching television, lying down to rest, and traveling in a vehicle. Scores for each item range from 0 (no chance of napping) to 3 (high probability of napping). The total score for Epworth Sleepiness Scale is based on a scale of 0 to 24, with a score ≥11 indicating [29].

### 2.6. Laboratory Findings

The following laboratory assessments were carried out: biochemical, metabolic, and nutritional status of patients; complete blood count; kidney and liver function; thyroid-stimulating hormone; HbA1c; albumin; calcium; phosphorus; ferritin; vitamin B12; folate; and vitamin D (25-hydroxy D3). All the biochemical tests were analyzed by using the Diagnostic Modular Systems (Roche E170 and P-800) autoanalyzer.

### 2.7. Serum Magnesium Level

Hypomagnesemia with serum magnesium level below 1.6 mg/dL and those with 1.6–2.6 mg/dL were considered normomagnesemic [18]. If serum magnesisum level was >2.6, it was accepted as hypermagnesemia, and older patients with hypermagnesemia were excluded.

### 2.8. Statistical Analysis

Descriptive statistics of categorical variables were reported as frequencies and percentages, while descriptive statistics of quantitative variables were given as mean, median, standard deviation, minimum and maximum values. Pearson chi-square was used to compare group ratios of categorical variables. The conformity of quantitative variables to normal distribution was examined with the Kolmogorov–Smirnov test. The assumption of homogeneity of variances was tested with the Levene test. In the mean comparison of two independent groups, *t*-test (Student *t*) was used in independent groups. The Mann–Whitney U test was used for the median comparison of two independent groups. In order to examine the multivariate effects of variables on hypomagnesemia, the variables that were significant in univariate analyzes and considered to be clinically significant were added to the binary logistic regression model as independent variables, and odds ratio values were obtained. These variables were hypertension, diabetes mellitus, coronary artery disease, Parkinson’s disease, hemoglobin, HDL cholesterol, HbA1c, triglyceride, and number of drugs used; 95% confidence intervals are given for odds ratio values. The Backward LR (likelihood ratio) method was used as a variable selection method in the model. Model explanatory power was examined with the Nagelkerke R square value, and the fit of the model was examined with the Hosmer and Lemeshow test. The statistical significance level was taken as 0.05 in the calculations and IBM SPSS Statistics for Windows, Version 26 (IBM Corp, Armonk, NY, USA) was used.

## 3. Results

A total of 938 older patients were included in the study. The mean age of the sample was 81.1 ± 7.6 years and 70.3% were female. The prevalence of hypomagnesemia, insomnia, and EDS was 14.3%, 54.1%, and 21.1%, respectively.

The comparison of the characteristics of the patients with hypomagnesemia and normomagnesemia is shown in Table 1. While the presence of EDS, hypertension, diabetes mellitus, and coronary artery disease was more common, Parkinson’s disease was less common in the hypomagnesemia group than the normomagnesia group (*p* < 0.05). There was no difference in insomnia between the two groups (*p* > 0.05). Hemoglobin and HDL cholesterol were lower, whereas HbA1c, triglyceride, and number of drugs used were higher in the hypomagnesemia group compared to the normomagnesia group (*p* < 0.05) (Table 1).

In both univariate analysis and multivariate analysis adjusted for gender, age, and all confounders, there were significant associations between hypomagnesemia and EDS [odds ratio (OR):1.7, 95% confidence interval (CI):1.6–2.6, and OR:1.9, 95% CI:1.2–3.3, respectively (*p* < 0.05)] (Table 2). While there was a relationship between diabates mellitus and hypomagnesemia in both univariate and multivariate analysis, age was significant only in multivariate analysis (*p* < 0.05). There were no significant relationships between hypomagnesemia and insomnia (*p* > 0.05).

## 4. Discussion

In this study, the frequency of hypomagnesemia in the elderly patients admitted to the outpatient hospital was 14.3%, in whom the frequency of hypertension, diabetes mellitus, and coronary artery disease was higher compared to those with normomagnesemia, but the frequency of Parkinson’s disease was lower. Triglyceride and HbA1c levels were higher, while hemoglobin and HDL levels were lower. In addition, drug use was higher in patients with hypomagnesemia. There was no relationship between insomnia and hypomagnesemia. EDS was present in one out of five older adults, the frequency of which was 1.9 times higher in those with hypomagnesemia than in those with normomagnesemia.

In our study, the prevalence of hypomagnesemia was 14.3%, which was lower than in other studies conducted in older people [15,16,17]. The possible reason for this may be that our study included older adults who were outpatients and were relatively healthy compared to those in other studies, and the cut-off values for hypomagnesemia somewhat differed between studies. For example, the prevalence was reported to be 24.3% in a study in which a value below 1.8 mg/dl was considered as hypomagnesemia and in hospitalized elderly people [15]. In another study using the same criteria for hypomagnesemia as the present study, but in medical settings including units such as geriatrics, oncology, and intensive care, the prevalence was 59.0% [17]. In the present study, frail patients such as moderate and advanced dementia patients were excluded, since ISI and ESS scales were required to be applied to the participants; this may account for the lower frequency of hypomagnesemia. In a similar study conducted in Türkiye where the same cut-off was used, but the mean age was 78 (81.1% in this study), hypomagnesemia was found to be 8.8% [30]. Magnesium intake, which may vary depending on geographical and ethnic differences, may also account for the differences in the prevalence of hypomagnesemia [31].

Magnesium is the second most important intracellular element after potassium in the cell. Magnesium activates more than 300 enzymes and is a co-factor of many enzymes, especially in carbohydrate metabolism [13]. For example, hypomagnesemia may cause insulin resistance by disrupting the function of the tyrosine kinase enzyme located in insulin receptors and increasing intracellular calcium, and, accordingly, disrupting blood sugar regulation and triggering oxidative stress [32,33]. Insulin resistance itself also leads to dyslipidemia; HDL decreases, while triglyceride increases [34]. In support of the previous literature, the present study identified a negative association between magnesium concentrations and HbA1c, which may be accounted for by decreased tubular reabsorption caused by hyperglycemia and/or hyperfiltration [33]. These above-mentioned mechanisms may explain why diabetes mellitus was higher, levels of HbA1c and triglycerides were higher, and HDL was lower in the hypomagnesemic elderly in our study. In addition, HT and CAD were also higher in those with hypomagnesemia. The reason for this may be that the positive effects of magnesium on endothelial function, regulation of vascular tone, regulation of catecholamine release, and renin angiotension aldosterone system are decreased in hypomagnesemics [32]. Another reason may be that the use of multiple drugs (for example, loop diuretics, thiazides, proton pump inhibitors, digoxin, some antidiabetics) may cause hypomagnesemia in patients with cardiovascular disease and diabetes mellitus [32,33,35]. According to our results, the number of drugs used in patients with hypomagnesemia was high. However, in our study, only the number of drugs was examined and individual drug groups could not be evaluated.

In this study, the main aim of which was to investigate the relationship between hypomagnesemia with insomnia and EDS, it was important to eliminate the influence of the factors mentioned above and other confounders, because factors such as diabetes mellitus, impaired blood sugar regulation, metabolic syndrome, CAD, multiple drug use, and anemia, which are excessive in hypomagnesemics, also affect sleep disorders [36,37]. Indeed, the present study demonstrated that hypomagnesemia was not associated with insomnia, but increased EDS by 1.9 times. However, results of studies investigating the effects of magnesium on sleep health (few of them carried out in the elderly) are conflicting [14]. For example, a cross-sectional study indicated that magnesium consumption was significantly higher in individuals with better sleep quality than in those with poor sleep quality [21]. In another cross-sectional study of 3304 female Japanese dietetics students aged 18–20 years, the midpoint of sleep was negatively associated with dietary magnesium intake [38]. However, the serum magnesium level of the participants in these two studies was not reported. This positive effect on sleep has not been demonstrated in randomized controlled trials. One study including 12 older adults (60 to 80 years) examined the relationship between oral magnesium supplementation and sleep. Magnesium was administered to the patients in 10 mmol doses and 20 mmol doses each for 3 days, and this was followed by 30 mmol doses for 2 weeks (i.e., 14 days). It was observed that wakefulness was reduced; however, this finding did not reach a level of significance [39]. Moreover, a recent crossover randomized double-blind placebo-controlled trial that included a total of 42 participants (average age 61.6 years) observed no effect on sleep disorders caused by nocturnal leg cramps when oral magnesium citrate was administered twice a day for one month [40]. In these studies, serum magnesium levels were evaluated neither at baseline nor after magnesium replacement. Therefore, it may have made no sense to replace magnesium in a normomagnesemic person initially. However, in a randomized controlled study conducted by Abbasi et al., they found that among the two groups in which there was no difference between serum magnesium levels at baseline, those who received magnesium replacement improved insomnia parameters in parallel with the increased serum magnesium level compared to placebo [23]. Nevertheless, it may be a limitation that EDS, which is one of the factors that frequently causes insomnia in the elderly, was not evaluated simultaneously in this study. In the present study, we showed that hypomagnesemia was associated with EDS rather than insomnia.

In a study similar to the present, Lai et al. showed that there was a negative correlation between serum magnesium levels and EDS in peritoneal dialysis patients; and in multivariate regression analysis, urinary magnesium was an independent predictor of EDS [41]. Previous evidence suggests that magnesium regulates sleep; because it acts as an NMDA and a GABA agonist, sleep architecture is closely associated with the glutamatergic and GABAergic system [20,39]. Indeed, the use of glutamatergic and GABAergic system modulators in the treatment of EDS has been of recent interest [42]. Moreover, healthy eating habits are impaired in the elderly with EDS, and thus those with EDS are at a higher risk of malnutrition [29]. Therefore, the elderly with EDS may have decreased magnesium intake and a higher frequency of hypomagnesemia. Additionally, EDS may lead to a reduction in leptin, an adiponectin that has been shown to decrease appetite. It is thus possible that there is a reduction in food intake owing to loss of appetite and skipped meals as a consequence of time asleep during the day; this may subsequently result in a higher risk of nutritional deficiencies [43]. Consequently, a bi-directional relationship between hypomagnesemia and EDS may occur. However, future studies are now required to test these hypotheses.

EDS, which is referred to as the condition of being sleepy during the day when an individual needs to be active and awake, is the second most common sleep disorder among sleep disorders, the importance of which has increased in recent years [44]. The decrease in the amplitude of the circadian rhythm with increasing age causes the frequency of night awakenings to increase [44]. Nonetheless, alterations in sleep physiology, such as decreased duration of slow-wave sleep (stages 3 and 4), increased compensatory stage 1 and 2 slow-wave sleep, and shortened rapid eye movement sleep, may give rise to EDS in older people [44]. For this reason, advancing age is a factor that increases the prevalence of EDS. Despite the fact that the prevalence of EDS between the ages of 30–60 is 11.0% in women and 6.7% in men, this rate increases in approximately 1 out of 3 people in both sexes over the age of 80 [45]. In our study, one out of every five elderly people was shown to have EDS and the rate was similar to the results of previous studies. The studies demonstrated that EDS was associated with adverse health conditions such as cognitive impairment, falls, sarcopenia, inability to perform activities of daily living, malnutrition, dysphagia, Vitamin D deficiency, depression, and cardiovascular events [29,46]. Even though EDS is associated with common and significant health problems, it is a condition that may be missed if it is not questioned in the evaluation of elderly patients in daily clinical practice. EDS is also common in neurodegenerative diseases, particularly dementia. Indeed, EDS is considered to be both an early indicator for future dementia and a risk factor. In our study, the determination of the relationship between hypomagnesemia and EDS suggests that magnesium replacement may also be beneficial in preventing negative health outcomes of EDS.

Findings from the present study must be interpreted in light of its limitations. First, the present study was cross-sectional in nature. Therefore, it is not known whether EDS leads to hypomagnesemia or vice versa. It is possible that the relationship is bidirectional. Second, self-reported scales were employed to assess EDS and insomnia, potentially introducing recall and social desirability bias into the findings; it would be prudent for future studies to employ objective measures of sleep, such as actigraphy. The strengths of our study are as follows: the number of adequate samples; evaluating both the presence of EDS and insomnia, and simultaneous evaluation of detailed comorbidities and comprehensive geriatric assessment parameters (e.g., nocturia, urinary incontinence, and functional status) which may affect sleep. To the best of the authors’ knowledge, this is the first study to evaluate hypomagnesaemia and EDS and insomnia simultaneously in older adults.

## 5. Conclusions

Hypomagnesemia is associated with hypertension, diabetes mellitus, coronary artery disease, low hemoglobin, and HDL cholesterol; and high HbA1c, triglyceride, and number of drugs used. Regardless of these factors, there was a significant relationship between hypomagnesemia and EDS in the elderly, but not insomnia. Therefore, hypomagnesemia or vice versa should be considered when evaluating an elderly patient with EDS. However, further longitudinal studies and intervention trials are needed to elucidate the complex pathophysiology of EDS and hypomagnesemia in older adults.

## Figures and Tables

**Table 1:** Characteristics of patients according to magnesium status.

	Hypomagnesemia	Normomagnesemia	*p* Value
Age, years	82.03 ± 7.42	80.96 ± 7.62	0.132 *
Female, (%)	73.9	69.8	0.359 **
Education, year	5 (0–28)	5 (0–24)	0.224 ***
***Comorbidities (%)***			
Hypertension	82.8	68.5	0.001 **
Diabetes Mellitus	68.7	32.7	0.001 **
Coronary Artery Disease	26.9	17.7	0.013 **
COPD	7.5	7.2	1.000 **
Cerebrovascular Events	11.9	10.4	0.650 **
Congestive Heart Disease	13.4	9.7	0.217 **
Peripheric Artery Disease	2.2	2.7	0.790 **
Parkinson’s Disease	3.7	9.3	0.043 **
Dementia	22.4	28.1	0.175 **
Osteoarthritis	21.6	16.9	0.220 **
***Laboratory Findings***			
Hemoglobin, g/dL	12.04 ± 1.52	12.56 ± 1.69	<0.001 *
HbA1c, %	6.5 (4.79–11.64)	5.95 (4.30–14)	0.001 ***
Ferritin, ng/mL	55.91 (4.31–1146.43)	57.55 (2.18–1897.39)	0.349 ***
Folate, ng/mL	6.45 (1.70–24)	6.6 (1.8–24)	0.909 ***
Vitamin B12, ng/mL	382 (95–2000)	371 (83–2000)	0.127 ***
Vitamin D, ng/mL	25.65 (5.30–97.40)	22.30 (3.90–118.9)	0.273 ***
GFR, mL/min/1.73 m^2^	60.64 ± 17.14	61.70 ± 18.96	0.524 *
Albumin, g/dL	4.2 (2.70–45)	4.3 (2.5–41.9)	0.486 ***
Triglycerides, mg/dL	137.5 (6–470)	120 (18–988)	0.034 ***
HDL cholesterol, mg/dL	12.04 ± 1.52	51.7 (17.9–102.7)	0.001 ***
LDL cholesterol mg/dL	6.5 (4.79–11.64)	128.1 (36.40–338)	0.134 ***
Calcium, mg/dL	55.91 (4.31–1146.43)	9.4 (7.6–12.6)	0.097 ***
Phosphorus, mg/dL	6.45 (1.70–24)	3.4 (1.5–8.7)	0.596 ***
TSH, mIU/L	382 (95–2000)	1.3 (0.01–26.15)	0.647 ***
***Comprehensive geriatric assessment***			
Urinary Incontinence, %	58.2	56.0	0.640 **
Nocturia episodes, number	2 (0–10)	2 (0–10)	0.101 ***
Number of drugs used	7 (0–15)	6 (0,25)	0.001 ***
Geriatric Depression Scale–15	4 (0–15)	4 (0–15)	0.382 ***
BADL	85 (0–100)	88 (0–100)	0.171 ***
IADL	13 (0–23)	14 (0–23)	0.101 ***
ISI	10 (0–28)	8 (0–28)	0.976 ***
Insomnia, %	56.7	53.6	0.514 **
Severe Insomnia, %	36.6	36.3	1.000 **
ESS	7 (0–24)	4 (0–24)	0.004 ***
EDS	%29.9	%19.7	0.009 **

BADL (Barthel Activities of Daily Living); COPD (Chronic obstructive pulmonary disease); EDS (Excessive Daytime Sleepiness); ESS (Epworth Sleepiness Scale); GFR (Glomerular filtration rate); HDL (High-Density Lipoprotein); IADL (Instrumental Activities of Daily Living); ISI (Insomnia Severity Index); LDL (Low-Density lipoprotein); TSH (Thyroid-stimulating hormone).* Student’s *t*-test was used. Descriptive statistics were given as mean and standard deviation.** Pearson’s chi square test was used.*** Mann–Whitney U test was used. Descriptive statistics were given as median (minimum–maximum).

**Table 2:** Predictors of hypomagnesemia.

	Univariate Analysis		Multivariate Analysis	
Parameters	OR, %95 CI	*p* Value	OR, %95 CI	*p* Value
Age	1.02 (0.99–1.04)	0.132	1.051 (1.014–1.089)	0.007
Female	1.23 (0.81–1.85)	0.336		
HT	2.21 (1.38–3.56)	<0.001	1.91 (0.97–3.75)	0.062
DM	4.49 (3.03–6.66)	<0.001	4.87 (2.82–8.42)	<0.001
CAD	1.71 (1.12–2.61)	0.013		
PD	2.65 (1.05–6.59)	0.038	0.380 (0.128–1.135)	0.088
TG	1.00 (1.00–1.01)	0.042		
HDL	0.97 (0.95–0.98)	<0.001	0.982 (0.96–1.001)	0.066
HbA1c	1.44 (1.24–1.67)	<0.001		
Number of Drugs	1.12 (1.07–1.18)	<0.001		
Hemoglobin	0.83 (0.74–0.93)	<0.001		
Insomnia	1.13 (0.78–1.64)	0.504		
EDS	1.74 (1.16–2.62)	0.008	1.96 (1.15–3.33)	0.013

Binary logistic regression analysis was used. The variables that were statistically significant in univariate analysis were included in the multivariate analysis. The backward LR variable selection method was used, and the final results were given in multivariate analysis. CAD: Coronary Artery Disease; CI: Confidence Interval; DM: Diabetes Mellitus, EDS: Excessive Daytime Sleepiness; HDL: High-Density Lipoprotein; HT: Hypertension; PD: Parkinson’s Disease; TG: Triglyceride. Nagelkerke R square value was obtained as 0.215. Hosmer and Lemeshow test *p* value was obtained as 0.250.

## Data Availability

The data that support the findings of this research are available from the corresponding author upon reasonable request.
